# Enhancing the antibacterial and surface hardness of glass ionomer cement modified with *Salvadora persica* and Chlorhexidine: An in vitro study

**DOI:** 10.12669/pjms.40.8.9190

**Published:** 2024-09

**Authors:** Adel Zia Siddiqui, Nighat Sultana, Zulfiqar Ali Mirani, Abdulaziz Abdullah Alkhureif, Faisal Rehan, Iftikhar Ahmed Siddiqui

**Affiliations:** 1 Adel Zia Siddiqui, MSc, B.D.S, CHPE Professor, Dept of Dental Material Sciences, Baqai Dental College, Karachi, Pakistan; 2 Nighat Sultana, Post Doc, Ph.D. Chief Scientific Officer, PCSIR Laboratories Complex, Karachi, Pakistan; 3 Zulfiqar Ali Mirani, Ph.D. Senior Scientific Officer. PCSIR Laboratories Complex, Karachi, Pakistan; 4 Abdulaziz Abdullah Alkhureif, Ph.D., MSc, B. Dent Tech Professor, Dental Health Department, College of Applied Medical Sciences, King Saud University, Riyadh, Saudi Arabia; 5 Faisal Rehan, M.Phil., BDS, BCom Lecturer, Centre for Rural Dentistry & Oral Health, School of Dentistry & Medical Sciences, Charles Sturt University, Australia; 6 Iftikhar Ahmed Siddiqui, PhD., M.Phil, MD, MBBS, MBA, CHPE Professor, Baqai Medical University, 51 Deh Tor, Toll Plaza, Super Highway, Gadap Road, Karachi, Pakistan

**Keywords:** *Salvadora persica* (*S. persica*), Glass ionomer cement (GIC), *Streptococcus mutans* (*S. mutans*), Vickers Hardness Number (VHN), Disc Diffusion Test (DDT)

## Abstract

**Objective:**

This laboratory study evaluated the effect of *Salvadora persica* (*S. persica*) root extracts and Chlorhexidine Digluconate (CHX) on the antibacterial and surface hardness properties of glass ionomer cement (GIC).

**Methods:**

The in vitro experimental study was conducted at the Baqai Institute of Pharmaceutical Sciences of Baqai Medical University, Karachi, Pakistan, from October 2022 to March 2023. There were a total four experimental groups. The first group consisted of ethanol extract (GIC-SPEE) and second group consisted of hexane extract as (GIC-SPHE) both prepared from *Salvadora persica* root respectively, and mixed with liquid of GIC separately. The third group comprised chlorhexidine (GIC-CHX) that was also mixing into liquid portion of GIC and the last group was Control i.e. (cGIC). The GIC samples were prepared by using stainless steel metallic moulds with dimension (5mm x 2mm), following the manufacturer guidelines. Antibacterial activity against *Streptococcus mutans* was done by disc diffusion test (DDT), and surface hardness test was done by Vickers hardness tester. Statistical analysis was performed using One-Way ANOVA and Tukey’s post hoc tests (p<0.05).

**Results:**

The antibacterial activity against *S. mutans* reported that the maximum zone of inhibition was obtained at 3 wt% by the GIC-SPEE, when compared with other experimental groups. For surface hardness, the highest mean and standard deviation and significant findings was reported by the group GIC-SPEE.

**Conclusions:**

Considering the outcome of this in vitro study, it can be concluded that the addition of 3 wt% GIC-SPEE increased the surface hardness and antibacterial activity against *Streptococcus mutans*.

## INTRODUCTION

Glass ionomer cement (GIC) is a popular dental biomaterial, known for its tooth-coloured appearance, chemical adhesion to teeth, fluoride release, and dentin-like coefficient of thermal expansion.^1^ Wilson and Kent pioneered the development of this cement, which was subsequently integrated into routine dental practice in 1972.^2^

Due to significant benefits for a range of dental uses, it is the perfect material for restorations since it chemically bonds to the tooth structure, negating the need for considerable dental preparation. GIC also helps to avoid secondary decay by releasing fluoride and recharging in the oral cavity. Because of this feature, GIC can maintain a stable fluoride content, serving as a reservoir for fluoride and so lowering demineralization process.^3^,^4^

Despite its advantages, GIC’s long-term survival rate is only around 65% over five years due to poor surface hardness and the development of secondary caries. The short term release of fluoride in GIC makes the tooth vulnerable to dental decay which prompted efforts to incorporate antibacterial agents without compromising its material properties.^5^

However, one study revealed that the inclusion of ZnO nanospherical and nanoflower particles resulted in a reduction in GIC’s surface hardness.^6^ Natural substances, like plant extracts, are gaining global popularity due to their accessibility, cost-effectiveness, and inherent biological properties. They are widely used in dental materials for applications such as preventing tooth decay, managing plaque, and promoting oral hygiene.^7^

*Salvadora persica* (Miswak) is a plant with benefits such as combating tooth decay and possess antibacterial, antifungal, and anti-inflammatory characteristics. Chlorhexidine (CHX), developed in the 1940s, is a broad-spectrum bisbiguanide antimicrobial used for controlling dental caries, gum inflammation, and plaque reduction. Therefore, this in vitro experimental study aimed to incorporate varying weight ratios of *S. persica* as well as CHX into a commercially available GIC (cGIC). The hypothesis was that *S. persica* and CHX would increase the antibacterial and hardness of cGIC.

## METHODS

This in vitro experimental study spanned a duration of six months, from October 2022 to March 2023. The preparation of *S. persica* root extract, antibacterial testing and surface hardness was carried out at the Pakistan Council for Scientific and Industrial Research (PCSIR). The GIC Samples used in this study were prepared at the Baqai Institute of Pharmaceutical Sciences, Baqai Medical University. *S. persica* roots were collected from Badin town, Sindh, and then were washed, dried, and grounded. The root powder was mixed with ethanol for two weeks. Ethanol crude extract was separated with Whatman filter paper. A Rotary Evaporator (Buchi Rotavapor, R-210, Singapore) machine was used at 70°C under vacuum for the collection of ethanol extract. Hexane was used for soaking root powder following the same protocol. The two extracts were then stored in a 4°C fridge to avoid degradation of the extract.^8^

### Ethical Approval:

This research project was reviewed and approved by the Institutional Review and Ethics Board (IREB) of Baqai Medical University, Karachi, with reference number: BMU-IREB/04-2023 on November 16, 2023.

In this in vitro study, GIC powder (GC Gold Label, Type II, Japan) was mixed with *S. persica* root extracts (ethanol: CAS No. 64-17-5, Grade: HPLC, Daejung Chemicals and Metals Co., Ltd, Korea., hexane: CAS No. 8032-32-4, Grade: HPLC Daejung Chemicals and Metals Co., Ltd, Korea.) and CHX: 20%, Lab On, Karachi at 0 wt%, 1 wt%, 3 wt%, and 5 wt% concentrations. These additives were combined with GIC liquid following manufacturer’s instructions. The unset cement was placed in moulds, covered with mylar strips and a glass slide to prevent bubble formation. After setting, samples were placed in glass bottles with deionized water at 37°C for 24 hours.

Samples were wiped dry before the antibacterial and hardness testing. The experimental groups of the study were as follows: (A) cGIC-control (Conventional Glass Ionomer Cement), (B) GIC-SPEE (Glass Ionomer Cement-*Salvadora Persica* Ethanol Extract), (C) GIC-SPHE (Glass Ionomer Cement-*Salvadora Persica* Hexane Extract) and (D) GIC-CHX (Glass Ionomer Cement-Chlorhexidine Digluconate).

### Antimicrobial testing by using disc diffusion test (DDT):

Disc-shaped samples were prepared using split metallic moulds measuring (5mm x 2mm) (height x diameter). The culture density for *Streptococcus mutans (*ATCC® 25175, Microbiologics, USA) was adjusted to 0.5 McFarland standard. Muller-Hilton agar (MHA) was used for bacteria growth and was incubated at 37°C for approximately 18-24 hours in the presence of 5-10% CO_2_. Gram-positive *Streptococcus mutans* (*S. mutans)* was inoculated on the dried surface of the MHA plate by streaking a cotton swab over the entire Petri agar dish. All the samples of the GIC were then placed onto the agar plate with the help of sterile forceps. Agar plates were placed in the incubator that was set for 24 hours at 37°C. The antibacterial activity against *S. mutans* was determined by the formation of inhibition zones around the disc-shaped samples of GIC. At the end, the zones were measured by using the plastic ruler.^9^ The antibacterial activity was investigated at all concentrations but the group that showed the best result was further evaluated for hardness testing.

### Surface hardness testing by Vickers hardness tester:

Disc-shaped samples were prepared using acrylic moulds with the following sample dimension (5mm x 2mm) (height x diameter). The test was carried out using a diamond indenter with a 50 g load applied to the sample. The indenter was in contact with the sample for 15 seconds (dwell time). The smooth surface of the test sample was placed on the test bench, and three indentations were made on the samples surface. A mean VHN value was calculated.^10^

### Statistical analysis:

The data were obtained by using IBM SPSS ver. 26 (IBM, New York, USA). Descriptive statistics were used to report the mean and standard deviation. One-Way ANOVA was employed, followed by Tukey’s post hoc tests. p-value of 0.05 or less was considered significant.

## RESULTS

**Fig. 1:**
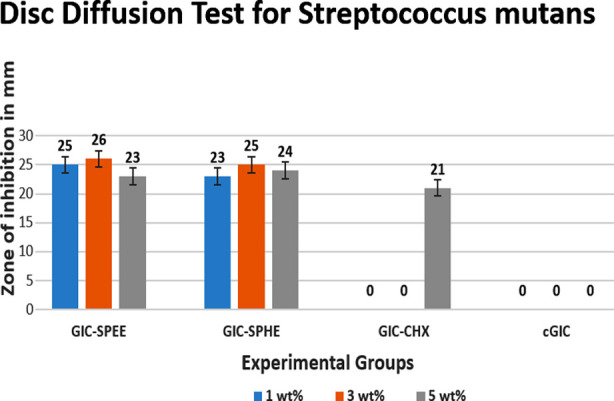
Disc Diffusion Test showing results of GIC-SPEE, GIC-SPHE, GIC-CHX and cGIC.

In this study, *S. mutans*, a gram-positive bacterium, was used to test antimicrobial activity. Results showed increased efficacy but decreased effectiveness at higher concentrations. GIC-SPEE had the largest inhibition zone at 3 wt% (26mm). GIC-SPHE showed its maximum inhibition zone at 3 wt% (25mm) and decreased at 5 wt%. GIC-CHX showed no inhibition zone at 1 wt% and 3 wt% but had a 21mm zone at 5 wt%. Compared to cGIC (control), the experimental groups exhibited activity. The results of the antimicrobial are displayed in the Table-I and Fig.1

**Table-I:** GIC-SPEE, GIC-SPHE, GIC-CHX and cGIC with varying concentrations as 0 wt%, 1 wt%, 3 wt%, 5 wt% and resulting in zone of inhibition for Streptococcus mutans.

Streptococcus mutans (Zone of inhibition in mm)			

Experimental Groups	Concentrations		

	1 wt% Mean SD	3 wt% Mean SD	5 wt% Mean SD
GIC-SPEE	25 ± 1.41	26 ± 1.41	23 ± 1.41
GIC-SPHE	23 ± 1.41	25 ± 1.41	24 ± 1.41
GIC-CHX	0	0	21 ± 1.41
cGIC	0	0	0

**Fig.2:**
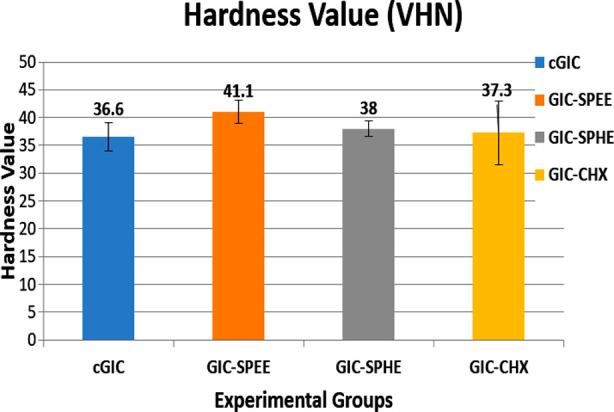
Values of hardness of all the experimental groups.

The current study investigated the Vickers hardness values of different groups related to GIC with the addition of 3 wt% of root extract and CHX. The means and standard deviation (SD) of Vickers hardness value (VHN) for each group cGIC (36.60 ± 2.59), GIC-SPEE (41.10 ± 2.08), GIC-SPHE (38.00 ± 1.41) and GIC-CHX (37.30 ± 5.72) is displayed in Fig.2. The study found that GIC-SPEE had significantly higher Vickers hardness values compared to the other groups. This suggests that the addition of 3 wt% of root extract to GIC-SPEE resulted in a significant increase in hardness compared to the cGIC and the other experimental groups GIC-SPHE and GIC-CHX as shown in Fig.2 and Table-II.

**Table-II:** Mean and Standard Deviation of (A) cGIC, (B) GIC-SPEE, (C) GIC-SPHE & (D) GIC- CHX showing Vickers hardness values (VHN) of all experimental groups.

Experimental Groups	Mean with Standard Deviation	p-value
cGIC	36.60^b^± 2.59	0.027
GIC-SPEE	41.10^a^± 2.08	
GIC-SPHE	38.00 ± 1.41	
GIC-CHX	37.30 ± 5.72	

## DISCUSSION

The hypothesis of this study for GIC-SPEE was accepted for antibacterial and hardness tests. This study modified the GIC with *S. persica* root extract and CHX to assess antibacterial activity and surface hardness. Medicinal plants are recognized for their diverse chemical compounds with antibacterial properties and therapeutic potential. Natural products, including plant extracts, are cost-effective and have low toxicity, gaining recognition as efficient alternatives for preventing dental caries.^7^

In a research study it was reported that *S. persica* alcoholic solvents have greater antibacterial activity then aqueous based solvents. Hence, in this current study solvents of ethanol and hexane, separately, were used to soak the root powder. Other constituent of *S. persica* extract, benzyisothiocyanate and isothiocyanates, both exhibits antibacterial potency. *S. persica* root extract has antibacterial activity due to presence of flavones, flavonoids and oleoresins in hexane extract.^11^

We investigated the antimicrobial activity against *S. mutans* using the DDT method. *S. mutans*, a key cause of tooth decay due to its role in carbohydrate fermentation and acid production.^12^ The agar disc diffusion method, developed in 1940, offers advantages like cost-effectiveness, simplicity, and versatility for testing various microorganisms.^13^

In our research, we examined GIC-SPEE, GIC-SPHE, and GIC-CHX experimental groups at 1 wt%, 3 wt%, and 5 wt% concentrations and compared them to the control group, cGIC. Studies by Singer et al. found that the use of high percentages of plant extract led to increased antimicrobial activity^13^ whereas, Tatari et al. used methanolic extract of *S. persica* root and noted no significant difference in antimicrobial activity between 2 wt% and 4 wt% concentrations that was aligned with our study.^14^

In another research examined methanol and water-based *S. persica* root extracts on *Staphylococcus aureus*, *Lactobacillus acidophilus*, and *Streptococcus mutans*, confirming antibacterial effects in both extracts.^15^ The study by Abhary et al. found that hexane-based *S. persica* extract had a smaller inhibition zone compared to ethanol-based *S. persica* extract that was contradicting with our results.^16^

For GIC-CHX, no activity was seen at 1 wt% and 3 wt%, but a 21 mm zone of inhibition was observed at 5 wt%. This study aligns with González et al. (2020), who noted increased CHX percentages enhance antimicrobial activity, with significant effects starting at 10% CHX incorporation, and the highest inhibition at 15%. Other studies support this, showing CHX effectiveness depends on concentration, potentially impacting GIC’s physical and mechanical properties.^13^,^17^ In a different study, Turkun et al. reported that incorporating CHX into GIC effectively targeted *S. mutans*.^18^ Bellis et al. reported a lack of antibacterial activity in cGIC, that were consistent with our findings.^19^

In our study, 3 wt% GIC-SPEE showed the most substantial 26 mm inhibition zone against *S. mutans*, declining at higher concentrations. GIC-SPHE achieved its highest 25 mm inhibition zone with a 3 wt% *S. persica* extract addition that also decreased at higher concentrations.

Meanwhile, researchers employed *S. persica* ethanol and hexane extracts and discovered that, ethanol-based extracts exhibited greater activity than those in hexane. The variation in the zone of inhibition could be due to the different pattern of diffusion exhibited by the extracts in the agar media. In the current study, the ethanol-based *S. persica* root extract led to a reduction in growth of bacteria compared to hexane-based extract. These results are in consistent to research done by Balto et al. 11 The reason that many of the antimicrobial compounds within plant extracts are relatively non-polar, they do not diffuse effectively within the aqueous agar matrix used in agar diffusion studies, thereby influencing the zone of inhibition.^20^

The key consideration here is that GIC-antibacterial combinations must possess optimal surface properties to withstand occlusal loads. Combining antibacterial materials with GIC can alter their physical properties, that is a well-documented fact. Researchers have strongly recommended that achieving appropriate modifications in the powder-to-liquid ratios, with a focus on creating well-balanced antibacterial concentrations, can help GIC antibacterial compositions maintain physical properties comparable to those of additives free GIC’s while still providing effective antibacterial features. Therefore, the preparation of GIC antibacterial combinations should be carried out carefully to effectively reduce bacteria without compromising the longevity of the restoration.^21^

Surface hardness is a crucial property that provides valuable insights into how well a dental material can withstand localized deformation.^22^,^23^ GIC-SPEE had the highest hardness (41.10±2.08) compared to cGIC (36.60±2.59). GIC-SPEE also showed greater hardness than GIC-SPHE and GIC-CHX (38.00±1.41 and 37.30±5.72, respectively), but this difference was not statistically significant. The presence of air voids during cement manipulation may limit GIC’s fracture resistance by initiating stress, acting as stress raisers, and deteriorating hardness.^24^

However, the experimental study presented here demonstrated an increase in hardness, possibly due to the presence of phenolic compounds and fatty acids. These components in *S. persica* react with the GIC and undergo a chelation reaction, resulting in the crosslinking of the powder. This, in turn, leads to a material with greater density and fewer voids within the set cement. This increase in surface hardness aligns with the findings of a study by Altunsoy et al. in 2016.^25^

In future research, it would be appropriate to use GIC-SPEE in 3 wt% concentration and evaluate its mechanical properties. Additionally, it is advised to check long term antibacterial activity against other cariogenic bacteria such as *Streptococcus sanguis*, *Actinomyces viscosus* and *Lactobacillus salivarius*.

### Limitations:

This in vitro experimental study has some limitations. Firstly, only *Streptococcus mutans* and one brand of glass ionomer cement GC Gold Label, Type II, Japan were used. Hence, for further work, different brand of restorative glass ionomers and other caries causing organisms should also be investigated. Secondly, the modified GIC did not undergo the aging procedure for surface hardness and antibacterial activity, which may be considered as a limitation. Therefore, to evaluate the durability and state of GIC, aging should be incorporated into future studies. Thirdly, this study was performed under in vitro conditions. Clinical conditions involve oral cavity affected by various oral environments such as saliva and variations in pH. Thus, these factors should also be assessed.

## CONCLUSION

Hence it can be concluded that the addition of 3 wt% of *S. persica* root extract in GIC-SPEE increased the surface hardness and antibacterial activity against *S. mutans*. However, the addition of CHX in GIC did not show antibacterial activity at low weight concentrations. The surface hardness of GIC-SPHE and GIC-CHX showed results that were nearer to the cGIC.

### Authors’ Contribution:

**AZS** did data collection, and manuscript writing, final approval and responsible for integrity of work.

**NS and AAA** did review and proofread the manuscript.

**ZAM** did data collection and antibacterial test.

**IAS** Conceptualization and supervision of the study.

**FR** analysed data and did statistical analysis.
